# Increasing arterial compliance by laser modification of fibro-calcific plaques

**DOI:** 10.3389/fcvm.2025.1652529

**Published:** 2025-10-15

**Authors:** S. Rajebahadur, N. Velluva Rayaroth, J. B. King, Yu. M. Alexandrovskaya, G. Guagliumi, A. Glatz, J. E. Johnson, F. Miranda Romero, D. Vela, V. M. Vinokur, T. E. Milner, E. N. Sobol

**Affiliations:** ^1^ Beckman Laser Institute and Medical Clinic, University of California Irvine, Irvine, CA, United States; ^2^ Biomedical Engineering Department, Samueli School of Engineering, University of California Irvine, Irvine, CA, United States; ^3^ Department of Surgery, Michael E. DeBakey Center for NanoBiophotonics, Baylor College of Medicine, Houston, TX, United States; ^4^ Terra Quantum AG, St. Gallen, Switzerland; ^5^ Interventional Cardiology, IRCCS Galeazzi Sant’Ambrogio Hospital, Milan, Italy; ^6^ Department of Physics, Northern Illinois University, DeKalb, IL, United States; ^7^ The Texas Heart Institute at Baylor College of Medicine, Houston, TX, United States

**Keywords:** arterial compliance, calcified plaques, laser, non-destructive modification, cardio-vascular disease

## Abstract

**Background:**

Calcium is a constituent of numerous types of atherosclerotic plaques. While various vessel modification devices have been introduced, plaque heterogeneity presents a challenge to direct therapy to specific components within the arterial wall.

**Aims:**

We introduce a novel approach for non-destructive modification of arterial fibrocalcific plaques with controlled spatial-temporal diode-laser irradiation. The laser thermomechanical approach enables the controlled formation of microstructural defects, stress relaxation, microcracking, and plaque molding. Primary objectives of this exploratory study include: (1) Determine an optimal laser dosimetry in fibro-calcific plaques in *ex vivo* human arteries that mitigates against non-specific thermal injury of the vessel wall; (2) Identify arterial structural modifications; and (3) Characterize changes in lumen, vessel diameter and compliance in response to laser irradiation.

**Methods:**

Diode laser radiation with a wavelength of 1,470 nm is delivered to *ex-vivo* human femoral artery specimens through an optical fiber inside a semi-compliant balloon containing heavy water. Laser dosimetry at the intimal surface is specified using a numerical model informed by vessel lumen diameter and beam profile measurements. Radiometric temperature increases at the outer surface of the vessel in response to laser irradiation is measured with an infrared camera. Micro-CT and IV-OCT images, recorded before and after laser irradiation, are aligned and co-registered using customized software. Micro-CT is utilized to identify changes in calcium plaques in response to laser irradiation (microcracking, voids, change in density). Changes in lumen diameter and compliance are assessed by High Frequency IV-OCT.

**Results:**

A candidate window for laser dosimetry is determined both theoretically and experimentally in the range of 150–300 W/cm^2^. Micro-CT images demonstrate fractures in calcium and changes in the plaque structure at irradiation sites with an azimuthal calcium extent greater than 300°. Increases in lumen area up to 28% and compliance up to 2.4x is observed.

**Conclusion:**

This proof-of-concept study demonstrated that modulated diode laser irradiation can modify the mechanical and structural characteristics of fibrocalcific arteries and increase vessel compliance. Additional studies are required in arteries with different levels of calcification and plaque distribution to optimize laser dosimetry for targeted vessel modifications.

## 1 Introduction

Atherosclerosis leads to a reduction of vessel compliance, increased stenosis, and vascular flow resistance. Atherosclerosis and plaque burden contribute to the progression of cardiovascular disease (CVD), the leading cause of death worldwide, accounting for 17.9 million deaths annually (1). The presence of calcification, a major component of advanced obstructed artery disease, is associated with the occurrence of Major Adverse Cardiovascular Events (MACE), including increased mortality, rate of acute myocardial infarction, stroke, and heart failure (2). Calcified plaques are resistant to conventional balloon angioplasty and stenting and can pose significant challenges during interventional and revascularization procedures (3). Successful treatment of fibro-calcific arterial plaques aims to restore lumen patency, increase vascular compliance and blood flow, and reduce the associated complications of interventional procedures.

Current percutaneous treatments for advanced calcified arteries include high-pressure balloon dilation, cutting or scoring balloons, intravascular lithotripsy (IVL), and mechanical orbital or laser atherectomy to prepare the lesion for additional stent implantation. Existing methods utilize a destructive, non-targeted approach to calcium and are associated with complications, primarily extensive vessel dissection and the potential for vessel perforation (4, 5). Shockwave Medical (Santa Clara, California) has introduced an electrical technique, currently widely used for treating calcified lesions. The Shockwave device generates large amplitude sonic waves (∼50 bar) that are transmitted through the balloon to the arterial wall, fracturing calcified plaque and facilitating further stent expansion of the artery at lower pressure (6, 7). Excimer laser atherectomy (ELCA, Philips) employs a UV laser (308 nm wavelength) to facilitate photoablation through various laser catheters. Radiation at a wavelength of 308 nm is absorbed by collagen, which can result in unintended damage to the vessel wall (8). These and other related complications underscore the importance of ongoing efforts to develop novel, safer, and more effective therapeutic modalities that target plaque precisely and modify vessels with minimal collateral damage.

For over four decades, laser irradiation of tissues has been employed clinically to ablate, coagulate, or otherwise modify targeted structures. Numerous clinical laser treatments rely on a hormetic effect, where the dose-response follows an Arndt-Schulz inverted U-shaped curve (9, 10). Non-destructive laser modification of tissue mechanical properties was first introduced to reshape or mold nasal septal cartilage (11). The mechanical elastic modulus of the irradiated cartilage is temporarily reduced by applying a specific laser dosimetry below the maximum in the associated Arndt-Schulz inverted U-shaped curve, allowing for cartilage reshaping in a user-specified manner (12). The non-destructive laser cartilage modification approach has been successfully applied in otolaryngology (13–16), in otoplasty to correct ear deformities (12), in orthopedics for restoration of intervertebral discs (16), and to treat meniscus cartilage in osteoarthritic knee joints (17). Laser reshaping of calcified costal cartilage has also been demonstrated (15).

We report the use of the thermomechanical effect of laser radiation as a novel approach for non-destructive modification of the structure and mechanical properties of multicomponent calcium arterial plaques. Controlled spatiotemporal modulation of laser radiation can induce the formation of microstructural defects, stress relaxation, microcracks, and calcified plaque molding. Simulations of thermal based therapies of arterial tissues that incorporate the coupled thermal, elastic, structural modifications have been reported (18–21). A model of tissue modification leading to a decreased mechanical elastic modulus has been developed in (22). The mathematical model describing these structural changes is based on two equations: a heat diffusion equation and a phenomenological equation for the phase field, which describes the modification and reduction of Young's elastic modulus through the breaking of chemical bonds. This model for one- and two-dimensional cases incorporates laser heating and tissue microcracking but cannot quantitatively predict the optimal laser dosimetry for the effective and safe laser molding of atherosclerotic plaques.

Here, we present a three-dimensional mathematical model that describes the non-stationary process of heat conduction during laser irradiation. The model predicts the spatiotemporal temperature distribution throughout the laser-irradiated vessel wall, the dynamics of bond breaking and microcracking in atherosclerotic plaques, as well as thermal non-specific damage to the vessel wall. The laser-based method can provide precise and selective modification of atherosclerotic plaques while minimizing damage to healthy tissue, thereby reducing risks such as vessel perforation and dissection.

The primary objectives of this study are: (1) To establish a therapeutic window for laser dosimetry in diseased *ex vivo* human fibrocalcific arteries that mitigate against non-specific thermal injury to the vessel wall; (2) To identify structural modifications in response to laser irradiation; and (3) To characterize changes in vessel and lumen diameter and compliance in response to laser irradiation.

## 2 Experimental methods and theoretical model

### 2.1 Protocol

**Figure 1:**
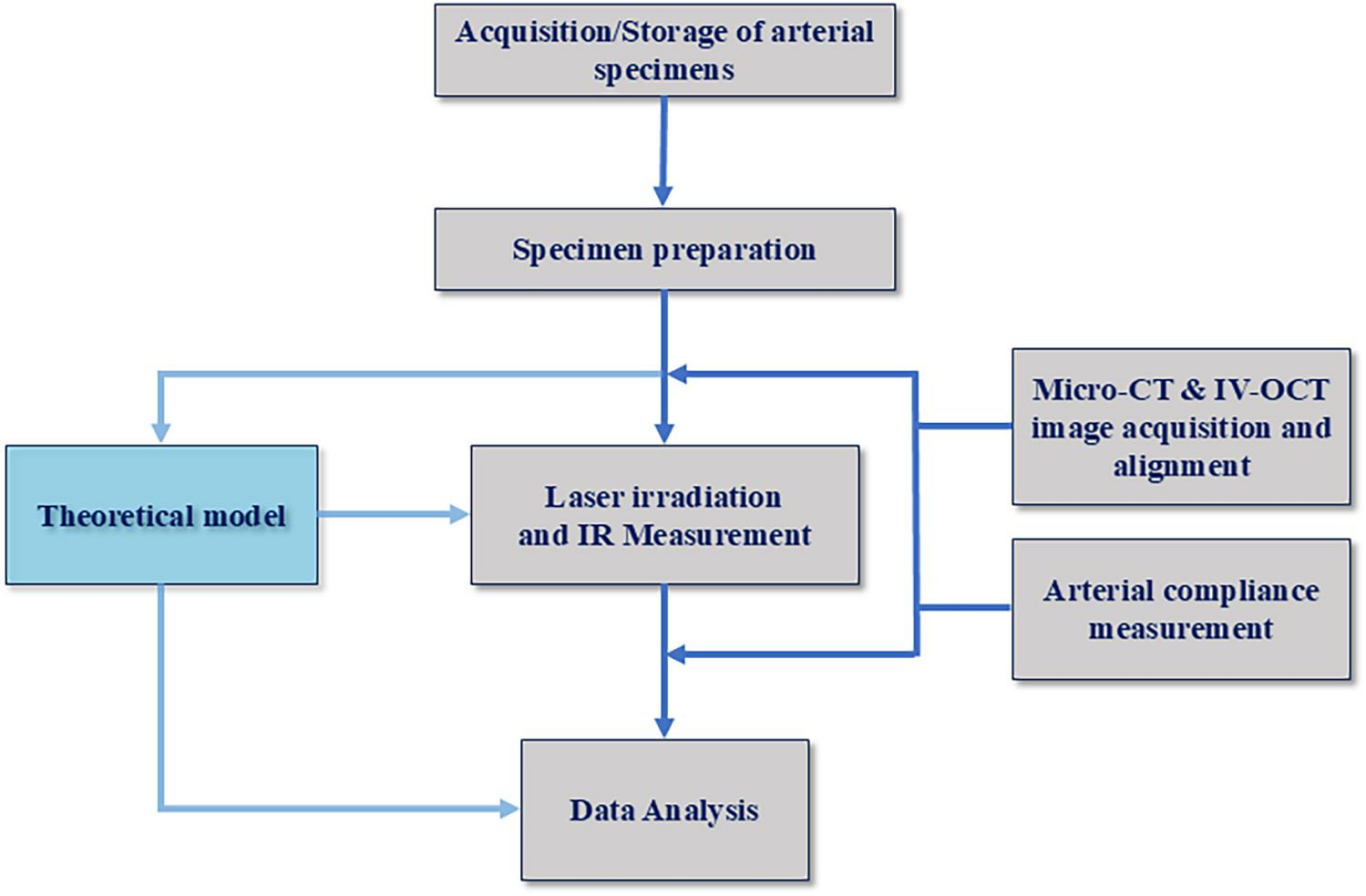
Protocol for laser arterial irradiation and analysis.

The multi-step experimental protocol begins with the acquisition of an arterial specimen and concludes with multi-modal image analysis, see Figure 1.

#### 2.1.1 Arterial specimen acquisition and storage

Human femoral arteries received from the University of California Irvine Willed Body Program were prepared inside a biosafety cabinet. After receiving the artery, a saline solution was gently irrigated over the outer arterial surface to clean and hydrate the specimen. The saline solution was also purged through the arterial lumen to remove any residual debris. Luer locks were fixed on proximal and distal positions of the artery using a cyanoacrylate fastener (super-glue). Femoral artery specimens were placed into a sterile container filled with chilled saline and stored at 4°C.

The characteristics of fibrous and calcium plaque included in the analysis as measured by Micro-CT and IV-OCT, are reported in Table 1. Plaque thickness ranges from ∼0.25 to ∼1 mm.

**Table 1:** Human femoral artery specimens included in the study.

Specimen number	Length (mm)	Wall thickness (mm)	Lumen diameter (mm)	Calcification arc angle
1	65	4	∼4.5	∼360°
2	65	4	∼4.5	∼317°
3	90	3	∼5	∼97°
4	90	3	∼5	∼102°

#### 2.1.2 Porcelain bead fiducial markers for micro-CT and IV-OCT imaging

Porcelain beads (1 mm diameter) are utilized as fiducial markers for micro-CT and IV-OCT imaging. Porcelain beads are carefully fixed onto the outer surface of the femoral artery at predefined longitudinal and azimuthal locations using a cyanoacrylate chemical fastener. The porcelain beads aid in identifying, registering, and differentiating regions in recorded Micro-CT and IV-OCT pre- and post-irradiation images.

### 2.2 Experimental setup for laser irradiation of arterial specimens

**Figure 2:**
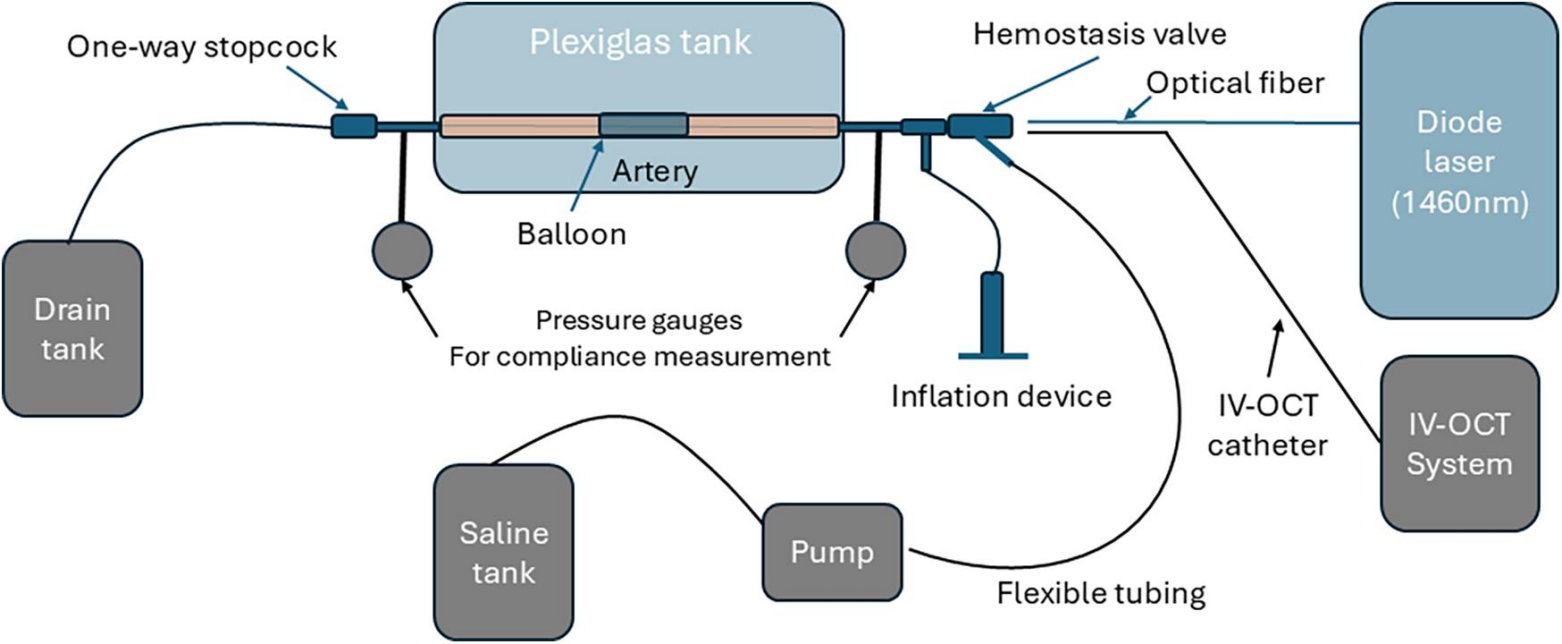
Schematic of experimental setup for laser irradiation and compliance measurement. The saline tank, pump, pressure gauges, and IV-OCT system were inactive during laser irradiation and used for compliance measurement.

A fiber-coupled diode laser source (nLight NL-p2-040-1470-10-A-R01) emitting light at a wavelength of 1,470 ± 10 nm at a maximum output power of 40 W is utilized. Diode laser light is delivered through a low-OH multimode optical fiber (NA = 0.22, 200 µm diameter core). Treatment laser light (1,470 nm) is combined with an aiming beam (633 nm) using a dichroic, see Figure 2. Both treatment (1,470 nm) and aiming (633 nm) beams are coupled into a 200 μm low OH multimode optical fiber that interfaces with a fiber-optic-rotary-junction (FORJ, Princetel, JSM). The FORJ allows the treatment fiber to rotate mechanically during irradiation while the proximal fiber remains stationary. An in-house angle-polished side-firing fiber (Med Fiber) with a 600 μm core diameter (NA 0.22) was used for the delivery of laser light. The distal end of the angle-polished side-firing fiber is encased in a sealed Poly-micro tube to ensure total-internal reflection from the cleaved surface. Laser dosimetry for irradiation using an angle-polished fiber utilizes a peak power of 15 W, a pulse duration of 10 ms, and a sequence of single-pulse emissions.

A plexiglass tank (23 cm × 16 cm × 16 cm, wall thickness 5.5 mm) with inlet and outlet ports is utilized to position the arterial specimen for laser irradiation. The femoral artery specimen was positioned in the plexiglass tank, ensuring the vessel was submerged in a sterile saline solution to mimic physiological conditions. Barbed leurs at the proximal and distal ends of the arterial specimen are inserted into polymer tubes that are mechanically fastened to the walls of the plexiglass tank. A semi-compliant balloon sized for the femoral artery specimen is inserted into the artery after sterilization.

The laser treatment fiber (angle polished side-firing fiber) is inserted into the artery while using the aiming beam to identify target tissue irradiation sites based on recorded Micro-CT images. The laser power is adjusted according to the arterial lumen diameter (measured via Micro-CT and IV-OCT) to deliver the specified laser dosimetry (e.g., power, pulse duration). Selected laser power (W) was determined from the specified laser dosimetry value (J/cm^2^), calculated using the measured beam profile and the distance from the central axis of the laser treatment fiber to the arterial lumen wall.

Just before laser irradiation, saline is partially removed from the plexiglass tank to expose the top surface of the arterial specimen. A FLIR camera (FLIR A320, Teledyne FLIR) is positioned at a fixed conjugate to the outside surface of the arterial wall. After the FLIR camera recording is initiated, emission from the 1,470 nm laser is triggered to irradiate the targeted area of the arterial specimen. Radiometric temperature monitoring of the arterial wall outer surface during laser irradiation was recorded using the FLIR camera.

### 2.3 Micro-CT and IV-OCT imaging

Micro-CT and IV-OCT imaging are conducted before and after laser irradiation. Pre-treatment Micro-CT facilitated the identification of candidate irradiation sites, the thickness of calcium in the arterial wall, wall thickness, and lumen diameter. Dimensions derived from Micro-CT of the arterial specimen are used to plan laser dosimetry and interpret results. A Siemens CT system (Siemens Inveon® Multi-Modality System) with resolutions ranging from 6 to 16 microns was employed to image the specimens; sample scan dimensions are approximately 25 mm × 17 mm. Inveon post-processing software provided advanced 3D viewing capabilities and cross-sectional views of the arterial specimen.

IV-OCT imaging was performed using a High-Frequency Intravascular Optical Coherence Tomography (HF-IV-OCT Gentuity) system. The IV-OCT and Micro-CT imaging and measurement procedures were performed both before and after laser irradiation of the treated arteries, and the results were compared.

#### 2.3.1 Pre- and post-irradiation IV-OCT and micro-CT image alignment

Customized software was developed to align the pre- and post-irradiation IV-OCT and Micro-CT images. Pre- and post-irradiation IV-OCT image data were exported from the IV-OCT system. Each pair of pre- and post-irradiation videos are imported into custom software for registration and alignment. For longitudinal alignment of IV-OCT images, the live views of the artery recorded during each pullback are used. In the live view, porcelain registration markers are used to measure small differences in pullback velocities between pre- and post-treatment. When time-stretching is necessary to achieve longitudinal alignment, the video of the shorter artery is slowed down, resulting in sparse frame duplication rather than lost frames.

For axial alignment of IV-OCT pullbacks, recorded images are used for reference. The alignment process involves: (a) identifying features in both pre- and post-irradiation IV-OCT images and rotating one video such that both features align at the same clock-position in both videos; (b) repeating this process for multiple features across frames; (c) interpolating the rotation of the video across all feature alignments to ensure both videos remain rotationally aligned during in-between frames. After alignment, both videos were cropped to highlight the IV-OCT measurement sections and the frame number, lumen area, deduced lumen diameter value, and displayed side-by-side.

Micro-CT imaging data is exported in a video format. These videos do not contain a live view, and porcelain registration markers are used for longitudinal alignment of pre- and post-irradiation Micro-CT images that were cropped and then displayed side-by-side.

### 2.4 Theoretical model

We consider a tissue modification mechanism that leads to stress relaxation and microcracking of fibrous calcium plaques, based on the controlled rupture of chemical bonds. The corresponding mathematical model is based on coupled partial differential equations: (1) Heating effects are described by a non-uniform heat diffusion equation (18); (2) The elastic response due to non-uniform heating of different materials and local bond rupture is captured by a phenomenological phase field model described by the function *a*(*r*, *t*). The phase field indicates the density of unbroken bonds at space-time position (*r*, *t*) (22, 23). Thermal expansion and the impact on heat propagation is implicitly included in the phase field *a*(*r*, *t*). Specifically, the activation energy (*U_b_*, Supplementary Equation S4) includes the thermal expansion coefficient [*a*(*T*)] as indicated in Supplementary Equation S4A. The temporal evolution of the phase field is given by a first-order partial differential equation:

(1) $$\frac{\partial a(r,t)}{\partial t}=(a-a_{0})P_{b}(r,t)$$

with an Arrhenius probability $P_{b}(r,t)$ for breaking bonds given by

(2) $$P_{b}(r,t)=P_{b0}\exp\;\left(-\frac{U_{b}(r)}{k_{B}T(r)}\right)$$

Here $0<a_{0}\ll 1$ is a residual phase-field when bonds are broken, *T* is absolute temperature depending on time *t* and coordinate *r*, *k_B_* -Bolzman constant, $P_{b0}$ a probability coefficient, and $U_{b}$ the energy barrier for breaking bonds which depends on the strain and stress in the system. The rate of bound breaking can be controlled by changes in *T* and *U_b_*.

The heat diffusion and other equations used in the analysis are described in Section 3.3 in Supplementary Information. These dynamic equations are solved numerically on high-performance graphics processors.

#### 2.4.1 Laser dosimetry

For each human femoral artery specimen, laser dosimetry was determined based on the calcium distribution and other characteristics such as lumen diameter and wall thickness obtained from IV-OCT and Micro-CT measurements. Various laser settings are investigated, including pulse width, frequency, exposure time, and laser spot area, depending on the type of optical fiber applicator. The following laser irradiation parameters are used in the experiments: 50-ms pulse duration at a frequency of 5 Hz, delivered in two pulse sequences of 5 s each (i.e., 25 pulses per pulse sequence). Artery 1 was treated using a conical tip fiber (MF600/630-RN Radial Tip fiber, MED-Fibers Inc) and provided an annular incident beam profile on the arterial lumen with a laser power density of 153 W/cm^2^. Artery 2 was treated with an angle-polished side-firing fiber (MF600/660-HCN Angle polished fiber, MED-Fibers Inc) and provided a slightly elliptical incident beam profile on the arterial lumen wall with a laser power density of 200 W/cm^2^. As shown in Table 2, artery 1 had a total of two laser irradiation sites, and artery 2 had a total of six laser irradiation sites. For both arteries, a 5 mm diameter semi-compliant balloon (BYond Medical, CA) filled with heavy water and inflated to a pressure of 5 BAR during laser irradiation. Heavy water was used to pressurize the balloon as it is transparent to a laser wavelength of 1,470 nm, thus ensuring that desired laser dose could be delivered to the vessel wall. The balloon is inflated with heavy water that is transparent to radiation emitted by the diode laser (24) and is utilized in an FDA approved atrial defibrillation catheter (25).

**Table 2:** Laser irradiation sites and corresponding dosimetry.

Specimen	Longitudinal irradiation sites	Azimuthal irradiation sites	Total irradiation sites	Fiber applicator	Spot size diameter (mm)	Laser dosimetry (W/cm^2^)
1	2	1	2	Conical tip	∼1	153
2	2	3	6	Angle cleaved	∼1	200
3	6	6	36	Angle cleaved	∼0.8	50–300
4	5	6	30	Angle cleaved	∼0.8	150, 300

Arteries 3 and 4 exhibit moderate atherosclerosis. Arterial compliance measurements were conducted on both arteries before and after laser irradiation. Laser irradiation parameters: 50-ms pulse durations at a frequency of 5 Hz, delivered in three pulse sequences of 5 s each. As shown in Table 2, artery 3 received a total of 36 laser irradiation sites, and artery 4 had a total of 30 laser irradiation sites. Arteries 3 and 4 were treated using a side-firing fiber (MF600/660-HCN Angle polished fiber, MED-Fibers Inc. Artery 3 was treated with laser power density ranging from 50 to 300 W/cm^2^. For artery 4, the laser dosimetry alternated between 150 and 300 W/cm^2^ based on calcium deposition at the irradiation site. For arteries 3 and 4, a 5.5 mm diameter semi-compliant balloon was inflated with heavy water to a pressure of 10 bars during laser irradiation.

### 2.5 Arterial compliance measurement

Arterial compliance is a measure of the ability of an artery to expand the lumen area in response to an increase in pressure. For arterial compliance measurements, the same plexiglass tank utilized for laser irradiation of the specimens is employed as shown with grey color in Figure 2. Two pressure gauges were positioned at distal and proximal locations, and one variable flow pump transported saline into the artery. One tube connects the saline tank to the pump, and the other tube connects the pump to a luer at the proximal end of the arterial specimen.

The artery is connected to the plexiglass tank using barbed luers, which are connected to the proximal and distal ends of the artery. The IV-OCT catheter is introduced through the proximal end and positioned to image irradiation sites. The recorded IV-OCT pullback provides quantitative image data of the arterial cross-sectional area, which is utilized to determine arterial compliance.

To study the effect of laser irradiation on arterial compliance, pre- and post-treatment IV-OCT images are recorded. First, all valves are opened to remove any air from inside the artery and connecting tubes. The specimen is properly flushed with saline, and the distal stopcock is closed. Closing the distal stopcock allows for pressurizing the artery at a selected pressure. Each pressure gauge is turned ON and zeroed before starting the saline pump.

The first reading is recorded at ambient atmospheric pressure. At this pressure, the IV-OCT catheter is introduced, and a pullback is performed to record baseline vascular lumen areal data. For the second set of readings, the pump is turned ON until intraluminal pressure reaches 1 PSI (51.7 mmHg). An IV-OCT pullback is recorded while maintaining a 1 PSI (51.7 mmHg) intraluminal pressure. The procedure is repeated for 2 PSI (103.4 mmHg) and 3 PSI (155.1 mmHg). After recording the pre-irradiation compliance data, we proceed with laser irradiation of the artery. A second set of compliance measurements is repeated after laser irradiation. IV-OCT data is exported, and the software is analyzed to determine the luminal diameter and area for each longitudinal position. Pre- and post-irradiation arterial compliance measurements are aligned in accordance with the procedures outlined above for IV-OCT alignment. The arterial diameter and area for each IV-OCT frame are calculated. After alignment and data extraction, pre- and post-irradiation arterial compliance data are computed. Compliance (*C*) was measured as *C* = Δ*A*/Δ*P* and has units of mm^2^/mmHg or mm^2^/psi.

## 3 Results

### 3.1 Computed spatial-temporal temperature in *ex vivo* arterial specimens

**Figure 3:**
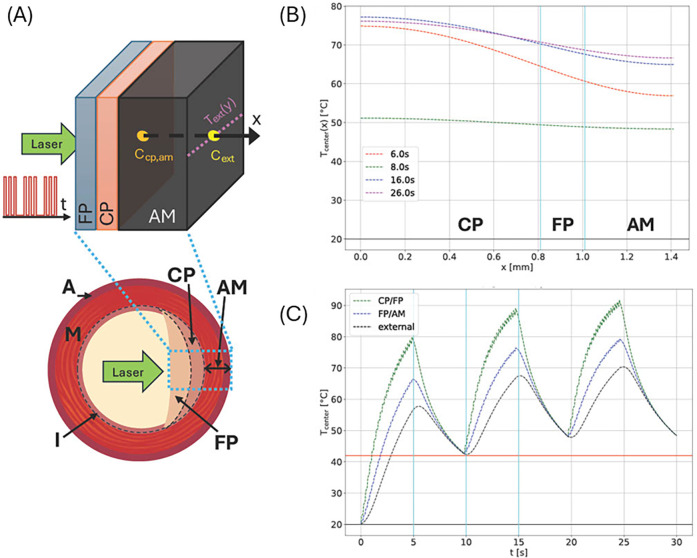
**(A)** Schematic cross section of an artery showing external adventitia (A), media (M), and intima (I) layers. In a diseased artery, the intima accumulates plaques—either calcified (CP) or fibrous (FP), as shown in the right half of the cross section. Diseased tissue is simulated by distinguishing CP, FP, and AM (the combined A and M layers) due to their different material parameters as an idealized plane layered system (top). Please note that plaque order and layer thicknesses may vary as indicated. Laser radiation enters the inside center, and the simulated laser pulse sequence and measuring points, and lines for temperature measurement are shown. **(B)** Temperature profiles along the central *x*-axis at different times for a system similar to site 1 of artery 3 with a CP layer of 0.81 mm and an FP layer of 0.2 mm between CP and AM. The laser power density was 300 W/cm^2^, and the circular laser spot had a radius of 0.5 mm. The pulse sequence consisted of a 5 Hz, 5 s-long sequence followed by a 5 s off period, repeated three times. **(C)** Simulated maximum temperatures at indicated layer interfaces (see vertical cyan lines in **(B)** and external boundary for the same irradiation site.

Arterial specimens are simulated in an idealized geometry in a cuboid volume, which is discretized in a regular mesh with a mesh length of 10 μm. The thickness of the arterial wall, including all plaque layers and adventitia and medium (AM), corresponds to the size in the *x*-direction, which is also the direction of incident laser radiation. The different arterial tissue components are implemented as homogeneous layers, stacked in the *x*-direction with a geometry corresponding to experimentally measured layer thicknesses using IV-OCT or Micro-CT (Figure 3A).

The perpendicular directions, *y* and *z*, correspond to the length and circumference of the artery, respectively. The dimensions in these directions are chosen sufficiently large enough such that thermal effects at the *y* and *z* boundaries can be neglected.

We simulate and compare the temperature evolution and distribution at the external boundary, specifically for arteries 2 and 3. Figure 3 shows the simulated layered structure and laser irradiation along the *x*-axis. The laser profile is depicted on the left. Panels (B) and (C) show temperature data corresponding to irradiation site 4 of artery 3, which were estimated from IV-OCT data. Panel (B) shows the temperature distribution along the *x*-axis (direction of incident laser radiation). Panel (C) shows the temporal temperature evolution at all internal tissue boundaries and the external surface.

### 3.2 Measured and computed radiometric temperature response profiles

**Figure 4:**
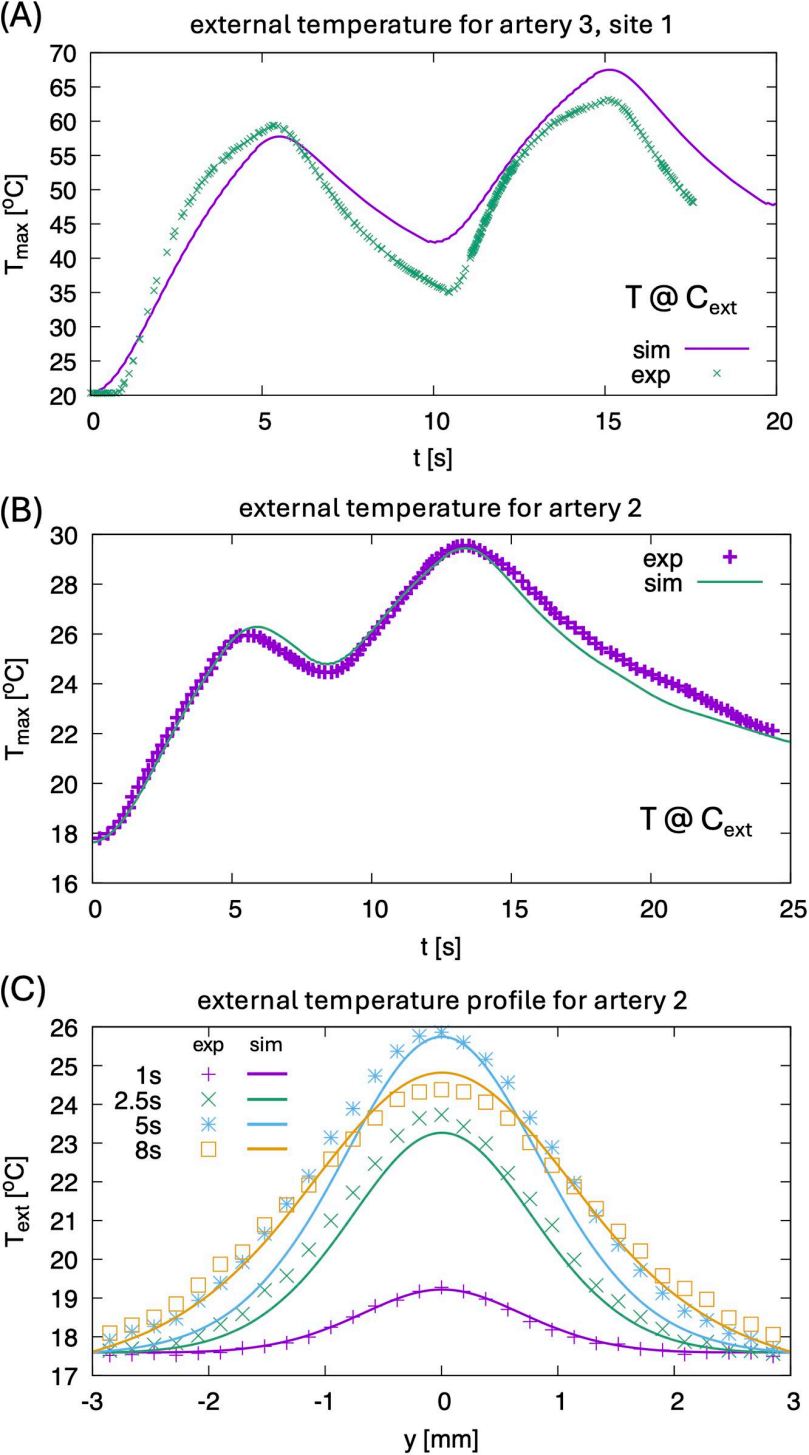
**(A)** Comparison of experimental maximum temperature at point c_ext_ for artery 3, site 1 for two pulse sequences, see also Figure 1C. Since the temperature inside the CP layer reaches 100°C, effects of latent heat and related strong evaporation lead to some differences in the heating and cooling rate between experiment and simulation. The latter does not consider the boiling of water. **(B)** Comparison of experimental maximum temperature at point C_ext_ for artery 2 (0.1 mm FP, 0.4 mm CP, 1.22 mm AM, power density 200 W/cm^2^, 0.3 mm × 0.45 mm elliptical laser spot) for two pulses. **(C)** Comparison of external temperature profiles for artery 2 at different times.

Figure 4 shows a representative example of measured vs. simulated radiometric temperature increase in response to laser irradiation. Simulated arterial layer thicknesses obtained from Micro-CT and IV-OCT are (CP, FP, AM): 0.81, 0.2, 0.99 mm, respectively. Measured radiometric temperature closely follows the simulated profile with maximum temperature at the outer surface reaching around 60°C. Figure 4B displays experimental vs. simulated radiometric temperature increase vs. time for artery 2 with laser dosimetry of 200 W/cm^2^. Simulated arterial layer thicknesses obtained from Micro-CT and IV-OCT are (CP, FP, AM): 0.4, 0.1, 1.22 respectively. The maximum temperature recorded on the outer surface of the artery is 30°C. Figure 4C demonstrates the temporal profile vs. position at different times.

### 3.3 Computed laser dosimetry and stress relaxation

To simulate complex heterogeneous diseased arterial tissues, tissue properties such as the heat diffusion constant are treated as spatially dependent functions—an important feature for discretization of the differential operators, see Supplemental Information.

Arterial specimens are simulated in an idealized geometry in a cuboid volume, which is discretized in a regular mesh with a mesh length of 10 μm. The thickness of the arterial wall, including all plaque layers and adventitia and medium (AM), corresponds to the size in *x*-direction, which is also the direction of incident laser radiation. The different arterial tissue components are implemented as homogeneous layers, stacked in the *x*-direction with a geometry corresponding to experimentally measured layer thicknesses using IV-OCT or Micro-CT (Figure 3A).

The perpendicular directions, *y* and *z*, correspond to the length and circumference of the artery, respectively. The dimensions in these directions are chosen sufficiently large enough such that thermal effects at the *y* and *z* boundaries can be neglected.

**Figure 5:**
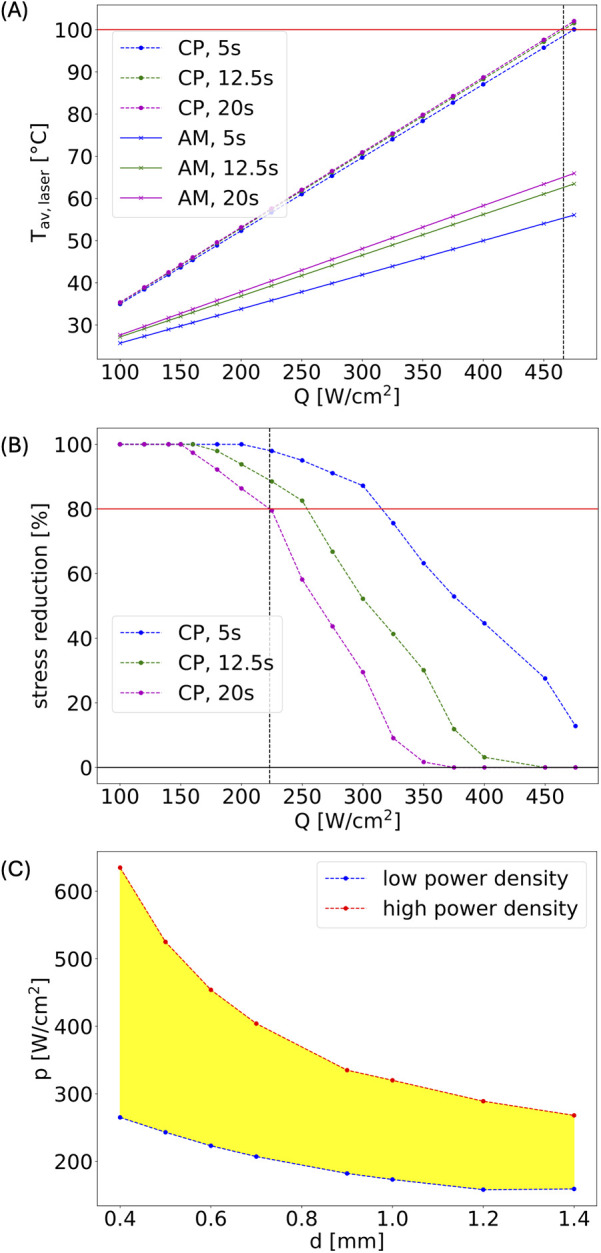
**(A)** Laser dosimetry window calculation for artery 2. **(B)** Calculated stress relaxation; laser fluence bounds for the therapeutic window are defined by a minimum of 20% stress relaxation (lower bound) or maximum temperature of 100°C in the CP (upper bound). **(C)** Therapeutic laser dosimetry window (yellow region) for a simulated system with 1.5 mm thick calcium plaque as a function of laser beam diameter *d*.

Figure 5 shows the dependence of the average temperature in CP and AM layers as function of laser power density at different times for a highly calcified system with 1.5 mm-thick CP layer. Panel (B) shows the corresponding dependence of the stress in the CP layer relative to the initial stress, obtained by a phase-field model which describes the temporal evolution of the specific density of unbroken chemical bonds, a(r,t), where *a* = 1 corresponds to no broken bonds and *a* = 0 to the case where all bonds are broken.

Using the unbroken bond density we can then derive the stress relaxation using the stress-strain relation, $\underline{\sigma }=E\underline{\varepsilon }$, with Young's modulus $E(r)=E_{0}\cdot a(r)+E_{1}$, where $E_{0}$ is the value if no bonds are broken and $E_{1}$ if all are broken—see Supplementary Information for more details.

Panels (A) and (B) correspond to a laser spot diameter of 0.6 mm. Defining the maximum safe power density for treatment as the power at which the CP layer reaches 100°C and the minimum power density at which the system shows a stress relaxation of 20% is taken as a therapeutic window. Performing the simulations for different laser spot sizes gives the range of estimated therapeutic windows, see panel (C).

**Figure 6:**
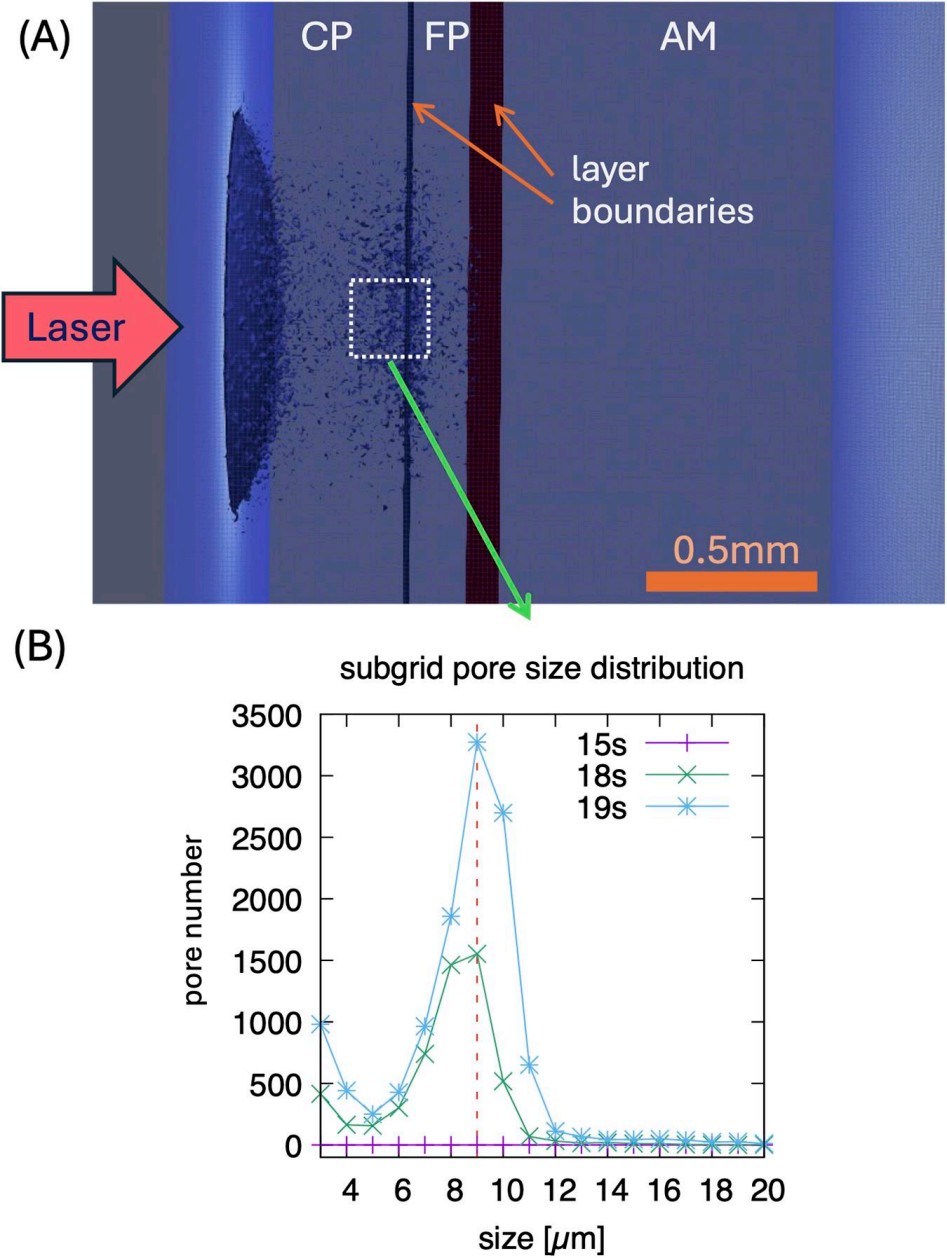
**(A)** Visualization of simulated pores/microcracks after 18 s laser irradiation on the 10 μm scale for site 4 of artery 3 (0.56 mm CP, 0.2 mm FP, 1.28 mm AM, power density 250 W/cm^2^, 0.5 mm circular laser spot. The shown pores represent averages on this length scale. **(B)** Distribution of pore sizes after 15, 18, and 19 s, showing a typical size of 9 μm in a sub-volume shown in **(A)**—in this sub volume, higher resolution simulations are performed for a 1 μm resolution. Note, typical pores form after about 15 s of irradiation.

The heat-diffusion calculations for the spatial-temporal temperature dependence are accompanied by the phase-field simulation mentioned above. Zones (areas) with 50% of broken chemical bonds are interpreted as microcracks. Figure 6 shows a visualization of this phase-field in the simulated layered simulation system after irradiation for 18 s at site 4 of artery 3. In the CP layer, laser irradiation produces microcracking, which is especially pronounced near the material interface layers, while no microcracks are visible in AM tissue. We analyzed the pore size distribution having typical size of 9 μm in a small sub-volume near the CP-FP interface (highlighted in panel (A) on the μm-scale, shown in panel (B).

### 3.4 Calcium plaque cracking

**Figure 7:**
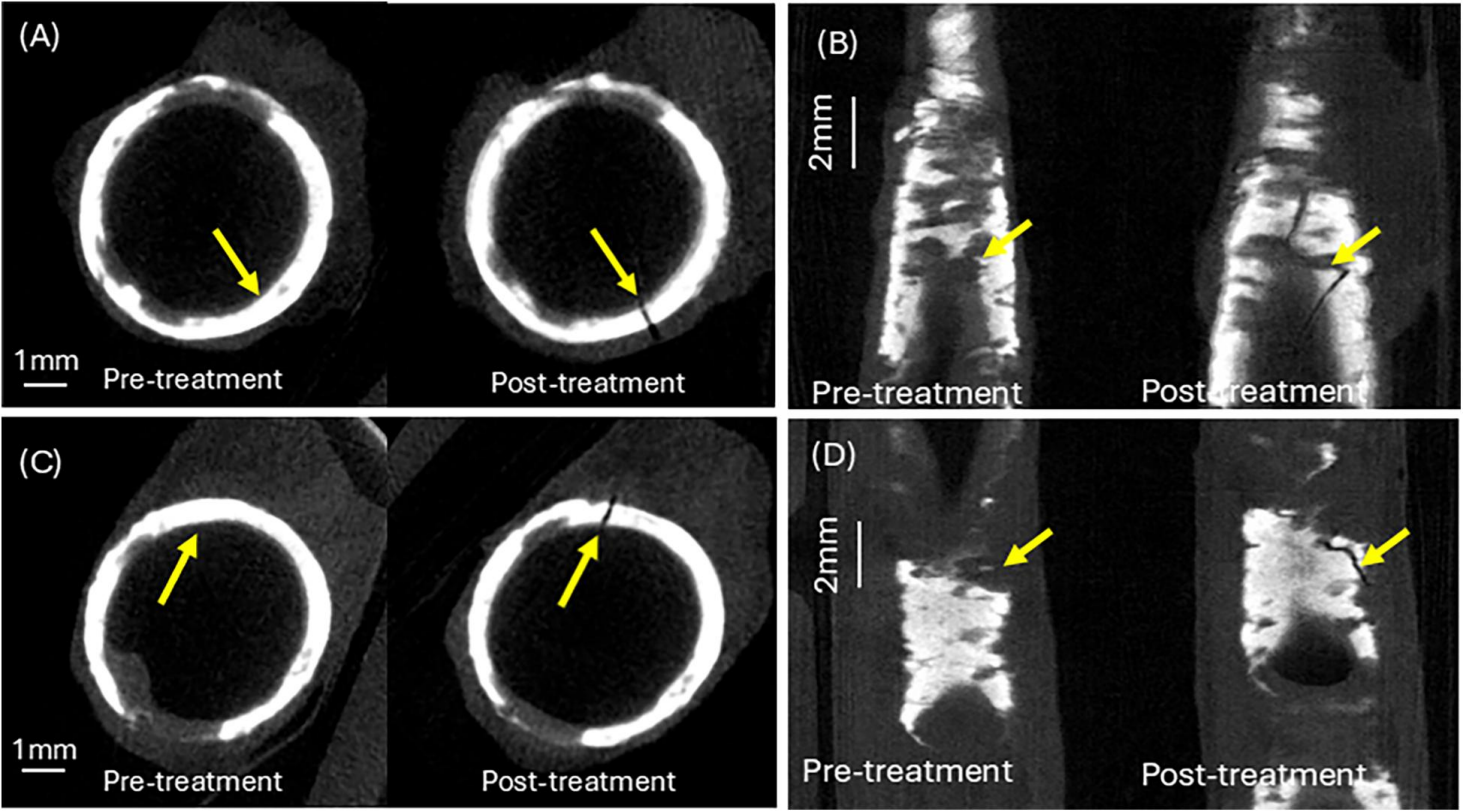
Calcium crack analysis of pre- and post–irradiation micro-CT images. **(A)** Calcium cracking at the laser irradiation site in artery 1. **(B)** Calcium cracking at the laser irradiation site in artery 2. **(C)** Top view of calcium crack at treatment site in artery 1. **(D)** Top view of calcium crack at treatment site in artery 2. See Supplementary Information for aligned Micro-CT pullback video.

A calcium crack width of greater than 1 mm was observed for both human femoral arteries. Figure 7C shows a top view of the calcium fracture in artery 1. The post-irradiation scan shows the presence of a large crack in calcium, measuring approximately 4 mm. Figure 7D shows a top view of the calcium fracture in artery 2. The calcium fracture in the post-irradiation scan is approximately 3 mm (Table 3).

**Table 3:** Measurement of average calcium thickness and calcium crack width and length.

Femoral artery specimen	Minimum calcium thickness (mm)	Maximum calcium thickness (mm)	Calcium arc angle (°)	Calcium crack width (mm)	Calcium crack length (mm)	Arterial lumen diameter (mm)
1	0.123	0.702	∼360	0.110	5.43	5.36
2	0.183	0.54	∼317	0.105	2.164	4.71

### 3.5 Structural and arterial compliance changes in response to laser irradiation

**Figure 8:**
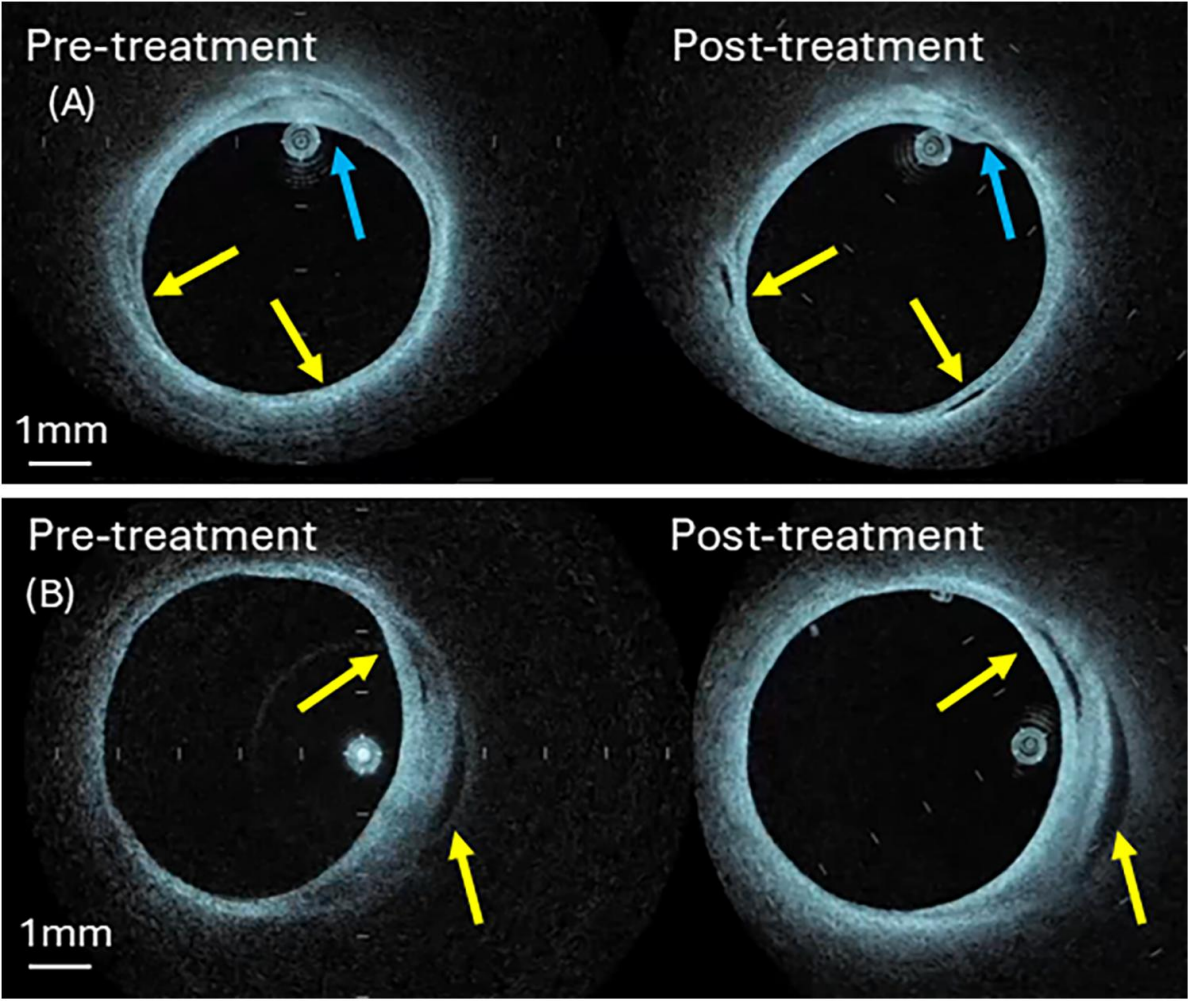
Pre- and post-irradiation IV-OCT B-scans at treatment sites **(A)** artery 3 **(B)** artery 4. Blue arrow identifies shrinkage of the fibrotic plaque components (panel **A**), while the yellow arrow shows new void formation (panel **A**) or expansion (panel **B**). See Supplementary Information for aligned IV-OCT pullback video.

Comparison of pre- and post-irradiation IV-OCT images showed laser-induced plaque modifications, see Figure 8.

Compliance measurement on two laser-treated human femoral arteries (Artery 3 and 4) is performed. Comparison of pre- and post-irradiation IV-OCT images reveals clear evidence of laser-induced modification, as shown in Figure 8. A post-irradiation image (Figure 8A) shows void formation in the arterial wall (yellow arrows). Similarly, we observe shrinkage in the arterial wall thickness, which is denoted by blue arrows. Figure 8B shows void expansion in the arterial wall in the post-irradiation image, which is denoted by yellow arrows. The voids and shrinkage of the irradiated tissue are signs of the accumulation and coalescence of microdefects formed due to the rupture of chemical bonds. These modifications in the arterial wall result in an increase in arterial compliance.

Post-irradiation images detect new void formation and shrinkage of plaque thickness in the arterial wall. The voids and shrinkage of the irradiated tissue may be signs of the accumulation and coalescence of microdefects formed due to the rupture of chemical bonds. These modifications in the arterial wall result in an increase in arterial compliance, see Table 4. A post-irradiation image shows the void formation, see Figure 8A, indicated by yellow arrows, and the shrinkage in the arterial wall, which is denoted by blue arrows. Figure 8B shows void expansion in the arterial wall in the post-irradiation image, which is denoted by yellow arrows.

**Table 4:** Pre- and post-irradiation IV-OCT measurements of arteries 3 and 4.

Specimen	Fluence at the treatment site	Pre-irradiation		Post-irradiation	
		Lumen area (mm^2^)	Lumen diameter (mm)	Lumen area (mm^2^)	Lumen diameter (mm)
3	250 W/cm^2^	14.9	4.4	16.2	4.5
4	300 W/cm^2^	20.6	5.1	21.6	5.2

#### 3.5.1 Arterial compliance measurements

Pre- and post-irradiation IV-OCT changes in arterial lumen area and diameter were measured by the longitudinally and azimuthally aligned pullbacks.

**Figure 9:**
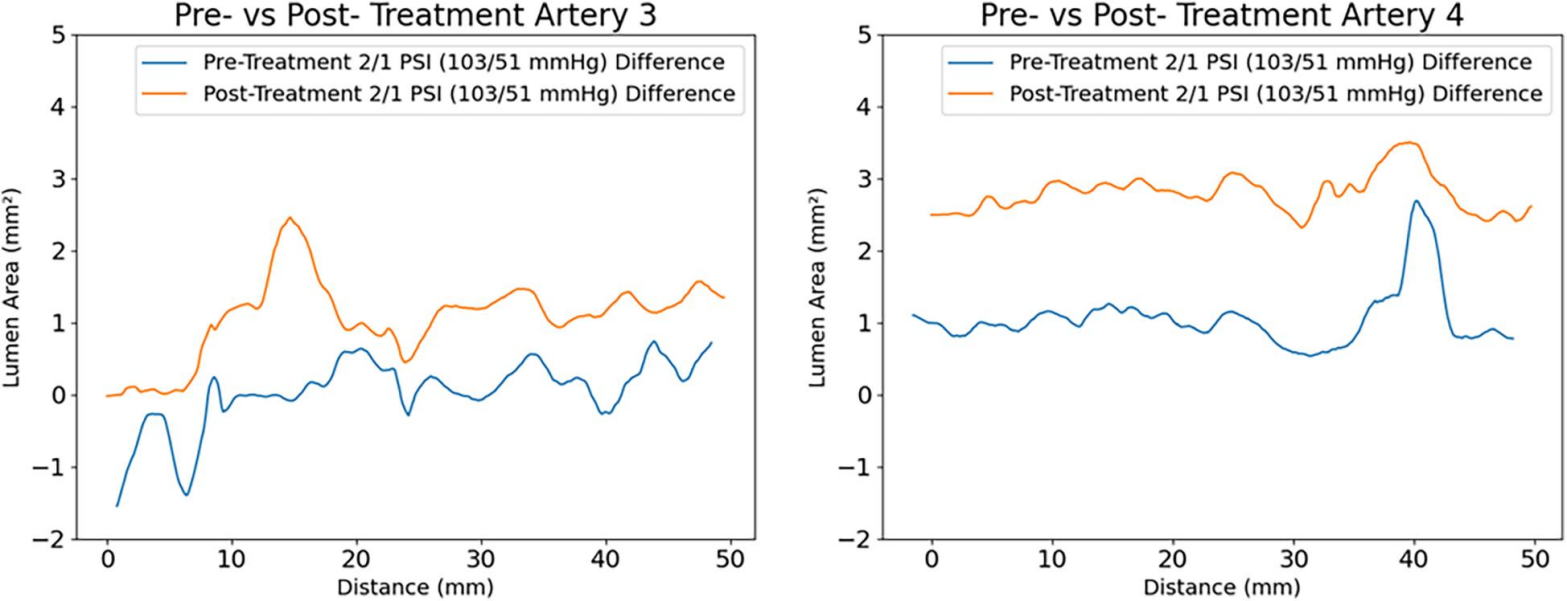
Measured compliance of the arteries at (2-1) PSI for artery 3 and 4 respectively. An average increase of lumen area of 1 mm^2^ with a maximum increase of 2.5 mm^2^ is observed in artery 3 (comparing pre -in blue- with post treatment- in yellow), with laser dosimetry that varied from 50 to 300 W/cm^2^. The largest increase in compliance is reported at the irradiation site corresponding to 300 W/cm^2^. A similar response of increased compliance over the entire irradiated region is displayed for artery 4.

An increase in lumen area was observed in all radiated arteries, of different degrees and location, depending on calcium distribution and specific laser dosimetry (varying from 50 to 300 W/cm^2^). An increase in compliance was also observed throughout the entire irradiated region, as shown in Figure 9.

## 4 Discussion

Current clinical calcium plaque modification techniques, including orbital/rotational atherectomy and intravascular lithotripsy and/or shockwave, rely on indirect mechanical or acoustic energy delivery that is not precisely targeted to specific plaque sites, nor can their dosimetry be dynamically controlled during irradiation. In contrast, the laser-based approach presented here offers some potential advantages of directed energy delivery to pre-imaged (IV-OCT) calcified regions through focused optical targeting, overcoming the spatial targeting limitations of conventional acoustic methods and customizable dosimetry that can be adapted in real-time to plaque morphology.

The present approach differs from other methods based on lithotripsy (destruction) processes, which utilize significant mechanical or thermal effects. Calcium plaques are modified using stress relaxation processes and the creation of microdefects (microcracks) that reduce the mechanical strength of hydroxyapatite (calcium plaques), thereby facilitating further expansion of the artery at a lower balloon pressure. The basis of tissue modification is the process of breaking chemical bonds, which occurs with laser heating and thermal deformation in heterogeneous calcified arteries. We rely on the large difference in thermal expansion coefficients between tissue water and hydroxyapatite (HA), where a small heating leads to thermomechanical strain and stress, resulting in the formation of microcracks at the boundaries of HA areas. For this purpose, we use infrared laser radiation absorbed not by collagen but by tissue water.

Thus, the novelty of the present approach lies in the targeted modification of the mechanical strength of calcium plaques and laser dosimetry (including temporal and spatial radiation and laser type) that allows us to achieve this goal. Specifically, the proposed technique has the potential advantage of targeting a specific diseased site in the artery and treatment with a controlled laser dosage.

The presented mathematical model is useful to better understand the effect and mechanism of laser-induced arterial modification. The model predicts spatiotemporal temperature distribution throughout the arterial wall under the influence of laser radiation. In addition, the dynamics of bond breaking and microcracking of calcified tissue, as well as the therapeutic window for safe and effective laser irradiation, are predicted.

Heating and structural modifications of tissues occur during laser irradiation and depend on the optical and thermophysical properties of the artery, the size and location of its structural components, laser parameters, and exposure time (18, 26). Primary laser dosimetry parameters include the wavelength, power, laser spot diameter, pulse duration, repetition frequency, the number of pulses in a pulse sequence, the number of such sequences, and the time interval between successive sequences.

The emission wavelength (1,470 nm) of diode laser radiation is absorbed by tissue water. Laser dosimetry includes a pulse duration of 50 ms, a pulse repetition frequency of 5 Hz that is selected based on preliminary calculations that consider both theoretical and experimental results of laser tissue modification in otolaryngology for correcting nasal septal deformities and shaping calcified costal cartilage implants (11–15). This application extends to otoplasty for correcting auricle deformities (12), orthopedics for restoring intervertebral discs (16), and also treating osteoarthritis of the knee joint.

To determine the optimal laser settings, the following laser parameters were varied: laser power, laser spot diameter, and exposure time.

Model results suggest:

(1) Calculations of the spatiotemporal temperature field for a calcified artery, which is considered as a multilayered structure, see Figure 3A, including calcium plaque CP, fibrous plaque FP, and the external part of the intact tissue (AM). This structure approximates the actual complex, heterogeneous structure of a calcified plaque, allowing for the study of the primary features of the laser's effect on a calcified artery, see Figures 3B,C. Note that due to the different optical properties of the various arterial constituents, the temperatures at the different boundaries between CP, FP, and AM differ significantly, see Figure 3C.
(2) Figure 4 illustrates a comparison of model results with experimental radiometric temperature data on the outer surfaces of arteries 2 and 3, supporting the validity of the mathematical model.
(3) Figures 5, 6 present the model's predicted results. Figure 5A illustrates the spatial distribution of microcracks along the various components of the calcified artery. Most microcracks are situated at calcified boundaries, and no microcracking in the AM layer of the vessel wall is anticipated. Figure 5B depicts the distribution of microcrack sizes. The typical sizes of microcracks range from 8 to 10 μm, which are difficult to detect using IV-OCT but sufficient to reduce the mechanical modulus of the calcium plaque. Microcracks emerge during laser irradiation, and their number increases rapidly 18 s after the onset of laser treatment. Similar findings regarding laser-induced microcracking have been previously examined and reported for cartilage and ocular tissues (13, 16, 22, 27, 28). The mathematical model enables the computation of internal stress reduction in the calcified artery due to the formation of microcracks, as shown in Figure 5. Figure 6C presents the therapeutic window for laser irradiation of calcified arteries as a function of laser spot size.

Note that the three-dimensional heat spread leads to greater energy losses and necessitates higher laser dosimetry for the smaller laser spot diameters used for arteries 3 and 4, as shown in Table 2. Microcracking occurs at lower power density for arteries 1 and 2 compared to arteries 3 and 4, due to reduced thermal energy losses associated with the larger spot diameter.

Although several processes can lead to non-specific tissue injury, including denaturation or cracking that may extend to the external vessel wall, we define an upper limit as the maximum laser fluence that will prevent water from boiling in the vessel wall. The mathematical model allows for the prediction of laser-induced heating, stress relaxation, and microcracking of arterial calcium plaques, as well as the therapeutic window for effective and safe laser irradiation of calcified arteries to enhance their compliance. The predicted therapeutic laser dosimetry window is sufficiently wide so that the proposed laser modification approach could be applied to coronary and peripheral arteries of various diameters. Further studies are required to translate the proposed laser approach into coronary arteries that have a higher risk profile.

Calcium in arteries is generally in the form of hydroxyapatite (29). Hydroxyapatite is considered a brittle material, suggesting that little or no plastic deformation occurs before the material is fractured. Cracking was predominantly observed experimentally in arterial segments with high circumferential calcium distribution (i.e., large arc angle), whereas non-calcified or minimally calcified regions remained intact. This aligns with studies demonstrating that calcium deposits alter stress distribution in arterial walls, creating localized mechanical discontinuities (30). In structural mechanics, preloading a brittle material is known to aid reaching the point of a critical load, a point beyond which the material fractures. Inflating the balloon before laser irradiation can aid in preloading the calcium plaque in the arterial wall and reduce the energy required to reach the critical load point. Studies report that calcified plaques fracture at lower pressures when pre-conditioned with mechanical strain (31). The laser's photothermal effect could synergize with this preload, exacerbating crack formation via thermo-mechanical induced fatigue.

While controlled cracking may facilitate plaque modification, variability in calcium distribution (arc angle, thickness) underscores the potential benefit of image-guided laser parameter optimization to avoid complications (e.g., dissections in non-calcified regions).

Laser irradiation induces localized circumferential expansion at laser irradiation sites, coinciding with regions of observed cracking.

Note that the formation of theoretically predicted micron-sized microcracks cannot be detected using IV-OCT, and the macrocracks shown in Figure 7 are the result of the coalescence of numerous small microcracks.

Although an increase in lumen area was observed in irradiated specimens, therapeutic efficacy can depend on multiple factors. For example, the wall shear stress impacts the hemodynamics in the artery and should also be considered. Moreover, in cases when the artery is stented after an interventional procedure, the preparation of the luminal surface should be carefully considered and appropriate imaging techniques (eg., IVUS or IV-OCT) employed (32–34).

Response of the arterial luminal wall to incident radiation was highly dependent on laser dosimetry. A table of compliance change for each treatment site is presented (Supplementary Table S3) in Supplementary Information. Although these preliminary data suggest that higher laser doses can result in larger increases in compliance, the trend is not monotonic and requires additional study. Considering the large heterogeneity in arterial specimen composition, the variation is not entirely unexpected. A higher laser dose corresponds to a higher risk of luminal damage. Achieving an optimal laser dosimetry and proper position on the Arndt-Schulz inverted U-shaped curve can ensure the desired therapeutic effect with minimal risk of arterial damage. Adjacent untreated segments also exhibited increased compliance, likely due to stress redistribution from the irradiation sites.

Experimental and model results suggest that optimal laser irradiation parameters must consider both calcium distribution and local wall thickness to achieve therapeutic benefits while minimizing long-term non-specific injuries. Future work can concentrate on computational modeling of stress redistribution and long-term *in vivo* studies of vascular remodeling following laser-induced mechanical alterations. While controlled cracking may facilitate plaque modification, variability in calcium distribution (arc angle, thickness) highlights the potential advantages of image-guided laser dosimetry optimization to avoid acute complications (e.g., dissections in non-calcified regions).

A potential advantage of the presented approach over existing methods using destructive lithotripsy processes is the non-destructive modification of tissue through controlled thermomechanical action, which provides stress relaxation and microcracks that facilitate further expansion of the artery at lower balloon pressures. The controlled introduction of microstresses in the arterial wall, however, could induce a pathogenic response. For example, the shear stress distribution along the luminal walls has long been associated with the pathogenesis of atherosclerotic plaques. Moreover, Jansen et al. (2024) caution that interactions between calcifications, collagen degradation, and macrophage activity can amplify inflammatory and thrombotic responses (35, 36). Therefore, any approach (such as that introduced in this work) to induce microcracks must carefully balance mechanical remodeling with biological consequences to avoid exacerbating plaque vulnerability (37).

The *ex vivo* studies conducted in this paper have obvious limitations, including the lack of experimental data on the safety of laser-assisted thermomechanical effects, the limited number of irradiated arteries with limited variability in plaque thickness and their spatial distribution. Although semi-compliant balloons were selected based on internal arterial diameter and the pressure inside of balloon was limited (4–7 bar), no analysis was completed to evaluate the differential effects of balloon angioplasty and laser induced micro-cracking. Side branches in the arterial specimens were closed with surgical stutters to ensure a complete seal. Thus, the arterial specimen could hold up the pressurization for compliance measurements.

The presented work is an early proof-of-concept study with a limited number of femoral artery specimens. An extended study with a statistically relevant sample size is required for further development and study to make recommendations in relation to the type and location (peripheral vs. coronary) of atherosclerosis that are candidates for therapy. Moreover, *ex vivo* experiments do not allow making conclusions about the long-term biological effects of laser irradiation. We have explored the basic principles of a new approach, the biological application of which will be evaluated in future *in vivo* and clinical studies.

There are several reasons why we expect the effectiveness of thermomechanical action on biological processes in calcified arteries.

Firstly, it is known that most biological cells, particularly arterial cells, are sensitive to external mechanical influences. The Nobel Prize in Physiology or Medicine 2021 was awarded to Ardem Patapoutian for the discovery of the mechanotransducers PIEZO1 and PIEZO2, which are activated by mechanical force and control cell function (38, 39). Modulated laser radiation enables precise control of the amplitude and frequency of thermomechanical action, thereby providing an effective tool for regulating cellular response and function.

Secondly, it is known that restenosis and recalcification in treated arteries are often associated with residual stress in the artery (40). Moreover, the presence of a calcium deposit creates local increases in stress, and peak stress in calcified arteries may increase risk of plaque rupture (41, 42).

Finally, the successful clinical application of our thermomechanical approach in spine surgery (16), orthopedics (17), otolaryngology (13), and ophthalmology (43) have demonstrated the stability of positive effects with at least a 5-year follow-up.

Therefore, our approach, including laser stress relaxation, should reduce the likelihood of recalcification, restenosis, and plaque rupture.

The presented laser intravascular approach might be advanced by incorporating light-based feedback control in an *in vivo* model, together with the development of machine learning algorithms.

## 5 Conclusion

A novel diode laser technique applied to fibrocalcific arteries induces non-destructive calcium modification of the plaque, resulting in increased vessel compliance and potentially serving as a minimally invasive method for enhancing blood flow. The recommended laser dosimetry, based on tested arteries, is specified for a range of incident laser spot diameters to provide stress relaxation and promote microcracks without causing untoward damage to the artery, especially in adventitia and media layers.

## Data Availability

The original contributions presented in the study are included in the article/Supplementary Material, further inquiries can be directed to the corresponding authors.
