# Supplementary material for: Increasing arterial compliance by laser modification of fibro-calcific plaques
Source: Front Cardiovasc Med. 2025 Oct 15;12:1652529. doi: 10.3389/fcvm.2025.1652529 (PMC12568616; doi:10.3389/fcvm.2025.1652529)
Supplement: Supplementary file 5 [file Datasheet1.pdf]

Supplemental information for

Increasing Arterial Compliance by Laser Modification of Fibro-Calcific Plaques

S. Rajebahadur[1,2,3], N. Velluva Rayaroth[1,3], J. B. King[1], Yu. M. Alexandrovskaya[4], G. Guagliumi[5], A. Glatz[6], J. E. Johnson[1, 3], F. Miranda Romero[1, 3], D. Vela[7], V. M. Vinokur[4], T.E. Milner[1, 2, 3], and E.N. Sobol[4]

Computational model for arterial plaque treatment

The mathematical model describing the laser treatment of the calcified arterial tissue is based on coupled partial differential equations: 1. Heating effects are described by the inhomogeneous heat-diffusion equation and 2. The elastic response due to inhomogeneous heating of the different materials and local breaking of bonds is captured by a phenomenological phase-field model describing the density of unbroken bonds evolving due to Arrhenius probabilities coupled to elasticity equations. Both are described to some detail in the following. These dynamic equations are solved numerically on high-performance graphics processing units.

Tissue heating due to laser irradiation

The spatial-temporal temperature evolution in a complex tissue system in response to laser irradiation is modelled using the inhomogeneous heat diffusion equation:

$$\rho(\mathbf{r})C_p(\mathbf{r})\frac{\partial T(\mathbf{r},t)}{\partial t} = \nabla \cdot [k_T(\mathbf{r})\nabla T(\mathbf{r},t)] + Q_s(\mathbf{r},\mathrm{t}), \qquad \text{(S1)}$$

where $\rho(\mathbf{r})$ is the density, $C_p(\mathbf{r})$ is the specific heat capacity, $k_T(\mathbf{r})$ the heat diffusion constant, and $Q_s(\mathbf{r},\mathrm{t})$ is the heat source (e.g., absorption of laser radiation), all are spatially dependent due to the inhomogeneity of the arterial composition. The spatial coordinates $\mathrm{r} = (x,y,z)$ are discretized on a rectangular mesh such that $\mathrm{r} \rightarrow \mathrm{r}_m = (ih_x, jh_y, kh_z)$, where m=$(i,j,k)$ are the coordinate (multi-)indices of mesh vertices. Important to note is that the first term of the right-hand-side of (S1) has, in general, two contributions $\nabla \cdot [k_T(\mathbf{r})\nabla T(\mathbf{r},t)] = \nabla k_T(\mathbf{r}) \cdot \nabla T(\mathbf{r},t) + k_T(\mathbf{r})\Delta T(\mathbf{r},t)$, which are crucial for the temperature distribution at internal material boundaries.

This allows us to describe, in general, any arbitrary shapes of, e.g., calcified or fibrous plaque tissues. Here we use the regular layer structure in the x-direction described in the main text using a mesh spacing of 10μm (see Figure 5A). The thicknesses of the layers are informed by OCT or micro-CT measurements. The lateral extension (in y- and z- directions) is chosen such that the effect of the laser heating is sufficiently far away from the boundaries to avoid finite size effects and periodic boundary conditions used. In x-direction, the boundary conditions are chosen as follows: at the irradiated, inner luminal surface cooling of the water-filled ballon is taken into account through the Fourier law and at the outer, external surface, a Neumann condition simulates a tissue-air boundary with passive convection. A typical simulation volume for a 5mm thick wall contains about $2 \cdot 10^7$ grid points and the heat diffusion equations are integrated with a timestep of 25μs for 10^6 steps corresponding to a real time of 25s.

For the heat source (Q_s) we use the following algebraic expressions

$$
\begin{aligned}
Q_s &= I_0 \kappa f(t) g(\mathrm{r}) \\
f(t) &= t/\tau_p \exp\left(-t/\tau_p\right) \\
g(\mathrm{r(x)}\ ) &= \exp[-(y^2+z^2)/r^2(x) - (k_a+k_s)x] \\
r(x) &= r_0 \exp(k_s x/2).
\end{aligned}
\qquad (S2)
$$

Here $f(t)$ represents the time dependence of the applied laser fluence, κ is tissue light absorption coefficient, and I_0 the laser fluence rate (W/cm^2).

Tissue modification and stress relaxation

In order to describe the breaking of, use introduce a phase field (PF) $a(\mathbf{r}, t) \in [0; 1]$, i.e. the specific density of unbroken chemical bonds. Here $a = 0$ means that all bonds at $\mathbf{r}$ are broken and $a = 1$ that all bonds are intact.

The temporal evolution of this density a is described [1] by

$$\frac{\partial a(\boldsymbol{r},t)}{\partial t} = -(a - a_0)P_b(\boldsymbol{r}, t) + \eta a, \qquad \text{(S2)}$$

with the Arrhenius probability, $P_b(\boldsymbol{r}, t)$, for breaking bonds

$$P_b(\boldsymbol{r}, t) = P_{b0} \exp\left(-\frac{U_b(\mathbf{r})}{T(\mathbf{r})}\right) \qquad \text{(S3)}$$

The energy barrier for breaking bonds is reduced by thermal gradients, ΔU_T, and elastic deformation, ΔU_d, described by the eigenvalues of the deformation matrix λ_i (U_0, γ_T, γ_d are phenomenological energy constants).

$$U_b(\mathbf{r}) = \mathrm{U}_0 - \Delta U_T - \Delta U_d \,. \tag{S4}$$

$$U_b(\mathbf{r}) = \mathrm{U}_0 - \Delta U_T - \Delta U_d = U_0 - \gamma_T|\nabla T(\mathbf{r},t)|^2 - \gamma_d(\lambda_1 + \lambda_2 + \lambda_3 - 3)\,. \tag{S5}$$

In (S2), $0 < a_0 \ll 1$ is a residual phase-field when bonds are broken, and $\eta \ll 1$ is introduced to counteract the natural denaturation of the tissue, which is the result of the finite energy barrier, U_0. This means the phase-field remains constant if not heating or deformation happens.

In (S3), P_{b0} a probability coefficient, and U_b the energy barrier (in units of Kelvin) for breaking bonds. This barrier is reduced by thermal gradients, ΔU_T, and elastic deformation, ΔU_d, with

$$\Delta U_T = \gamma_T|\nabla T(\mathbf{r},t)|^2 + \alpha(T)\,|\Delta T\,(\mathbf{r},\,t)|^2 \tag{S4a}$$

$$\Delta U_d = \gamma_d(\lambda_1 + \lambda_2 + \lambda_3 - 3). \tag{S4b}$$

The first term describes the effect of thermal gradients and promotes the breaking of bonds in regions with large temperature variations, typically at material boundaries. α(T) is the coefficient of thermal expansion, which increases significantly (from $2\text{x}10^{-6}$ to $7\text{x}10^{-6}$ 1/K) with increasing temperature from 20 to 100 °C for tissue water [2]. The second term, ΔU_d, describes local deformation of the material due to laser irradiation and, according to the Mooney-Rivlin theory [3,4], can be expressed by the eigenvalues of the deformation matrix λ_i:

$$\lambda_i = \frac{\partial r_i}{\partial r'_i} = 1 + \frac{\sigma_i}{E_0 a_i + E_1}$$

where i ∈ {1,2,3} for the three spatial directions and the prime indicates the reference coordinate system before deformation.

We assume that initial stress on the system ($\sigma = F/A$) is aligned with the main axes, i.e., the stress tensor is diagonal. Then we use the stress-strain relation to obtain the deformation of the system: $\underline{\boldsymbol{\sigma}} = E\underline{\boldsymbol{\varepsilon}}$, where E is the Young's modulus, $\underline{\boldsymbol{\sigma}}$ the stress tensor, and $\underline{\boldsymbol{\varepsilon}}$ the strain. Then, we write $E(\mathbf{r}) = E_0 \cdot a(\mathbf{r}) + E_1$, where E_0 is the value if no bonds are broken and E_1 if all are broken ($a = 0$). With

$$\boldsymbol{\varepsilon} = \nabla(\mathbf{r} - \mathbf{r}')\,,$$

we get the eigenvalues of the deformation matrix shown above.

With these eigenvalues, we get the energy barrier reduction (S4b) in three dimensions and the elastic energy density $\epsilon(\mathbf{r}) = (\lambda_1 + \lambda_2 + \lambda_3 - 3)/(6E)$. The parameters γ_d, E_0, E_1, and initial stress are given parameter – see table.

Table S1: Elasticity parameters for different tissue used in simulations.

parameter \ material	**AM**	**FP**	**CP**	**References**
probability, P_0	0.05	0.05	0.05	4
energy barrier, U_0 [K]	2100	2100	2100	2, 5
coefficient $\boldsymbol{\gamma_T}$ [μm^2/K]	3360	5460	6720	4, 5
coefficient, $\boldsymbol{\gamma_d}$ [K]	25	25	25	4, 5
Young's modulus E_0 [MPa]	1.0	0.3	1400	4, 6
Young's modulus E_1 [MPa]	0.1	0.03	140	6

The tissue-specific parameters for the fibrous plaque (FP), calcified plaque (CP), and combined adventitia and media intact tissue (AM) are summarized in Table S2. These include thermal parameters and laser absorption/scattering lengths.

Table S2: Tissue parameters used in simulations.

parameter \ material	**AM**	**FP**	**CP**	**References**
**density, ρ [kg/m^3]**	1200	1200	1500	5
**specific heat, c_p [m^2/(K s^2)]**	3800	3800	3400	5, 7
**heat conductivity, k_T [W/(mK)]**	0.5	0.5	0.5	5
**absorption coefficient, κ [1/m]**	3500	2600	2100	8, 9
**scattering rate, k_s [1/m]**	2050	1750	2250	9, 10
**absorption rate, k_a [1/m]**	3500	2600	2100	9 -11

Compliance Measurements

Below is the table representing average change in compliance and standard deviation with respect to treatment zones for arteries 3 and 4. Statistical Analysis is performed for different treatment sites corresponding to different laser dosimetry

Table S3: Average change in luminal area and standard deviation with respect to different treatment sites and laser dosimetry.

Specimen	Treatment site	Dosage (W/cm^2)	Mean change (mm^2)	Standard deviation
3	1	300	2.12	0.31
	2	50	0.67	0.43
	3	150	0.53	0.10
	4	250	0.97	0.23
	5	250	1.02	0.05
	6	100	0.82	0.10
4	1	150	1.82	0.02
	2	300	1.92	0.02
	3	300	1.89	0.08
	4	300	2.14	0.16
	5	150	1.80	0.30

References

1. Shnirel'man AI, Sobol EN, Bagratashvili VN. A new mechanism for stress relaxation in cartilaginous tissue upon laser heating. Laser physics. 2004;14(3):404-8.
2. . Chaplin M. Water structure and science, updated in **2022**, https://water.lsbu.ac.uk/water/water_vibrational_spectrum.html#ir2.
3. Mooney, M., 1940, A theory of large elastic deformation, Journal of Applied Physics, 11(9), pp. 582–592.
4. Rivlin, R. S., 1948, Large elastic deformations of isotropic materials. *IV. Further developments of the general theory*, Philosophical Transactions of the Royal Society of London. Series A, Mathematical and Physical Sciences, 241(835), pp. 379–397.
5. McIntosh, R. L., Anderson, V (2010). A Comprehensive Tissue Properties Database Provided For The Assessment Of a Human At Rest. Biophysical Reviews and Letters. 5(3): 129-151. https://doi.org/10.1142/S1793048010001184
6. Astala, R.; Stott, M.J. First-Principles Study of Hydroxyapatite Surfaces and Water Adsorption. *Phys. Rev. B* **2008**, *78*, 075427, doi:10.1103/PhysRevB.78.075427;
7. Posner, A.S.; Beebe, R.A. The Surface Chemistry of Bone Mineral and Related Calcium Phosphates. Seminars in Arthritis and Rheumatism 1975, 4, 267–291, doi:10.1016/0049-0172(75)90013-X.

8. Genina, E. Optical Properties of Skin, Subcutaneous, and Muscle Tissues: A Review. https://doi.org/10.1142/S1793545811001319
9. Çilesiz, I.F.; Welch, A.J. Light Dosimetry: Effects of Dehydration and Thermal Damage on the Optical Properties of the Human Aorta. Appl. Opt. 1993, 32, 477, doi:10.1364/AO.32.000477.
10. Jacques SL. Optical properties of biological tissues: a review. Phys Med Biol. 2013 Jun 7;58(11):R37-61. doi: 10.1088/0031-9155/58/11/R37

11. Keijzer, M.; Jacques, S.L.; Prahl, S.A.; Welch, A.J. Light Distributions in Artery Tissue: Monte Carlo Simulations for Finite-Diameter Laser Beams. *Lasers Surg. Med.* **1989**, *9*, 148–154, doi:10.1002/lsm.1900090210.
